# Carbon Nanomaterials in Agriculture: A Critical Review

**DOI:** 10.3389/fpls.2016.00172

**Published:** 2016-02-22

**Authors:** Arnab Mukherjee, Sanghamitra Majumdar, Alia D. Servin, Luca Pagano, Om Parkash Dhankher, Jason C. White

**Affiliations:** ^1^Department of Analytical Chemistry, Connecticut Agricultural Experiment Station, New HavenCT, USA; ^2^Department of Life Sciences, University of ParmaParma, Italy; ^3^Stockbridge School of Agriculture, University of Massachusetts Amherst, AmherstMA, USA

**Keywords:** carbon nano-materials, toxicity, soil-microbes, co-contaminants, CNM-plant interactions

## Abstract

There has been great interest in the use of carbon nano-materials (CNMs) in agriculture. However, the existing literature reveals mixed effects from CNM exposure on plants, ranging from enhanced crop yield to acute cytotoxicity and genetic alteration. These seemingly inconsistent research-outcomes, taken with the current technological limitations for *in situ* CNM detection, present significant hurdles to the wide scale use of CNMs in agriculture. The objective of this review is to evaluate the current literature, including studies with both positive and negative effects of different CNMs (e.g., carbon nano-tubes, fullerenes, carbon nanoparticles, and carbon nano-horns, among others) on terrestrial plants and associated soil-dwelling microbes. The effects of CNMs on the uptake of various co-contaminants will also be discussed. Last, we highlight critical knowledge gaps, including the need for more soil-based investigations under environmentally relevant conditions. In addition, efforts need to be focused on better understanding of the underlying mechanism of CNM-plant interactions.

## Introduction

Carbon nanomaterials (CNMs) are a class of engineered nanomaterials (ENMs) seeing increased applications due to their exceptional optical, electrical, mechanical, and thermal properties (Hurt et al., 2006; Bennett et al., 2013; Srivastava et al., 2015). The synthesis of CNMs has evolved significantly over the last two decades. The discovery of Buckminster (C_60_) fullerenes in 1985 was followed by carbon nanotubes in 1991, and the graphene-family in 2004, which continues to dominate most applications (Bergmann and Machado, 2015; Hong et al., 2015).

Carbon assumes thermodynamically favorable configurations from sp^3^ to sp^2^, depending on the heat of formation and pressure conditions, to produce structures such as nano-diamonds and graphene sheets (Mauter and Elimelech, 2008). CNMs combine the quantum effects at the nanoscale, the stability of resonance structures, and the tunable distinctive physico-chemical properties due sp^3^ character of C-C bonds (Mauter and Elimelech, 2008). The CNM family includes fullerenes, nano-onions, nano-cones, nano-horns, carbon dots, carbon nanotubes (CNTs), nano-beads, nano-fibers, nano-diamonds, and graphene (Sharon and Sharon, 2010; Cha et al., 2013; Baptista et al., 2015). They exhibit huge diversity in their structures as well as size, which is not exclusively confined to nanoscale (<100 nm) in all dimensions. For example, graphene is a two dimensional -one atom thick sheet of sp^2^-C-atoms in a hexagonal honeycomb crystal lattice, with sp^3^-C-atoms at the defect sites. It is regarded as the “building block” for other graphenic/graphitic nanoallotropes (Georgakilas et al., 2015). Fullerenes are hollow spheres with a hexagonal network of carbon atoms and ≤ 1 nm in diameter (Chichiriccò and Poma, 2015). Concentric multi-layered fullerenes called nano-onions are also seeing increased use in biomedical and electrochemical applications (Bartelmess and Giordani, 2014). Although they are limited in actual commercialization, Benn et al. (2011) detected pristine C_60_ fullerenes in different commercial cosmetics, at concentrations ranging from 0.04 to 1.1 μg/g. CNTs are cylindrical structures with open or closed ends and can further be categorized into single-wall (SWCNTs) and multi-wall nanotubes (MWCNTs) depending on the number of concentric layers of rolled graphene sheets (Yang et al., 2010; De Volder et al., 2013). The outer diameter of CNTs is typically 0.8 to 2 nm for SWCNTs, and 5–20 nm for MWCNTs (De Volder et al., 2013). The lengths range from 100 nm to several centimeters, depending on the desired application (De Volder et al., 2013). Owing to their tunable physical properties with respect to graphene layers, functionalization, and chirality, CNTs and associated polymer composites are the most produced CNMs, with applications including in electronics, optics, nanomedicine, and biosensors (Mauter and Elimelech, 2008; Yang et al., 2010; De Volder et al., 2013; Keller et al., 2013). They are also utilized for environmental applications such as solar cells, renewable energy production with higher efficiency, remediation purposes, detection/sensors for pollutants, and for contaminant degradation (Mauter and Elimelech, 2008; Baptista et al., 2015; Rasool and Lee, 2015). Additionally, hollow CNMs such as fullerenes, CNTs, and nano-horns are capable of serving as carrier molecules for metals, atoms or active ingredients in sensors and biomedical applications (Georgakilas et al., 2015).

Recent CNM exposure studies have demonstrated beneficial and stimulatory effects on plants *in vitro* or in culture conditions. These findings have increased interest in potential applications in agriculture and food production (Serag et al., 2015), although the findings are somewhat inconsistent. MWCNTs have been reported to be internalized in plant roots (Lin et al., 2009). SWCNTs have also been proven to efficiently cross the cell wall and membranes of tobacco cells upon *in vitro* exposure, with subsequent transport to specific cellular organelles; this could be taken as evidence for potential use as “nanotransporters” (Samaj et al., 2004; Liu et al., 2009). Additionally, the suppression of organic contaminant uptake by plants has been reported in the presence of select CNMs (Petersen et al., 2009; De La Torre-Roche et al., 2013).

In spite of the wide scope of CNMs application in agriculture, there are several limiting factors impeding their extensive use. First, although in artificial plant cultures select CNMs have rendered toxicity at physiological, cellular, and genetic levels (Ghosh et al., 2015), there are very limited number of studies evaluating the chronic phytotoxicity of CNMs under environmentally relevant conditions. Notably, assessment of CNM toxicity in soil is confounded by a large number of factors, including low solubility, reactivity with soil organic matter, and large background from the residual C in soil; all factors limit detection and complicate a mechanistic understanding of cellular interactions. Second, there is little information on the nutritional effects and potential genetic modifications/damages in CNM-exposed plants, including transgenerational impacts (Lin et al., 2009; Husen and Siddiqi, 2014; Ghosh et al., 2015). Third, stability of the CNMs in a complex media and residual leaching of attached metals or contaminants from CNMs (Bennett et al., 2013) is poorly understood. Finally, CNM transfer from soil to plants and subsequently to higher herbivore and carnivore trophic levels is completely unknown. As such, direct agricultural applications are still in experimental phase given this limited knowledge of CNM fate and effects on agroecosystems.

Interestingly, in spite of the above listed concerns, CNTs are among the 10 most produced ENMs (Keller et al., 2013), and along with graphene, CNTs are considered viable for expanded use in commercial applications (De Volder et al., 2013). With increasing scope of production and application, their release into the environment with subsequent human exposure is inevitable. Petersen et al. (2011) provided a comprehensive review on the environmental exposure, fate, and risks associated with the use of CNTs. A thorough understanding of the mechanistic interactions of CNMs with plants and associated microbial communities is necessary to evaluate the risk associated with widespread use in agriculture. The aim of the current review is to provide a thorough evaluation of the existing literature on CNM fate in soil, including effects on plants and associated soil microorganisms. We will also critically assess the limitations in the current literature and highlight topics worthy of future investigation.

### Fate of Carbon Nanomaterials in the Environment

In spite of growing interest and research, regulatory agencies have become concerned that the potential negative impacts of these materials in the environment may outweigh their benefits. Compared to application-based research and development, studies on the ecotoxicity of CNMs are quite limited and involve a narrow range of test species and materials, growth media, and analytical techniques. Owing to their unique and reactive properties, some speculate that CNMs may have the potential to not only impact individual species but also to disrupt ecological dynamics (Jackson et al., 2013). Accurate delineation of the sources, pathways and sinks is needed for a complete CNM risk assessment. In recent years, the global production of CNTs were reported to range from 55 to 3,300 tons and of fullerenes from 0.6 to 1620 tons (Sun et al., 2014). These materials can enter the environment via emission from manufacturing processes (Bello et al., 2009), either accidentally or/and as waste discharge in air/water/landfills.

Human exposure to CNMs may occur through occupational settings or through indirect exposure from various environmental matrices such as air, water, and soils/sediments. Nowack et al. (2013) suggested that the predominant release pathway is during initial synthesis and handling of ENMs, which will result in occupational exposure and perhaps large-scale environmental exposure if an accidental release occurs. Ogura et al. (2013) investigated the release characteristics of SWCNTs in a pilot scale-plant, using on-site aerosol measurements and dustiness tests with vortex shaking and transferring containers. The authors reported that the elemental carbon content increased during the synthesis, which also includes the particles from the combustion process. But the concentration of elemental carbon was within the suggested occupational exposure limits proposed by US NIOSH (7 μg/m^3^). Interestingly, dustiness testing suggested low release characteristics and low drop impact of SWCNTs owing to their low bulk densities; mostly micron-sized CNT clusters were observed by scanning electron microscopy (SEM). This might suggest that SWCNTs have lower chances of accumulating in soil and sediments. The major fraction of fullerene and CNTs produced are as polymer composites, thus making them a major source for release of CNMs. Petersen et al. (2011) discussed various release pathways of CNT-incorporated polymer nanocomposites, during their usage and disposal in a comprehensive review. The authors reviewed various natural conditions including radiation, UV light, moisture, temperature and microbes leading to degradation of polymer matrix holding the CNTs, resulting in particle release. Also, activities during composite such as mechanical abrasion and incineration can lead to release of the nanomaterials in an uncontrolled manner. However, the literature is largely silent on the release scenarios of many CNMs, including fullerenes, graphene, etc. and their fate in various environmental matrices.

Importantly, CNM stability and transport will not only depend on native material properties but also on the characteristics of any conjugated composites and on the surrounding conditions (Nowack et al., 2013). In the past few years, mass flow modeling studies have been implemented to assess the flow of various ENMs in the environment, starting from the global/ regional production to release via usage and waste disposal, and finally, compartmentalization in different ecological matrices (Gottschalk et al., 2009; Keller et al., 2013; Mueller et al., 2013; Sun et al., 2014). Employing Monte Carlo simulations based on total usage in a defined region (EU and Switzerland), Sun et al. (2014) suggested that most of the CNMs flow from the production to recycling and waste incineration plants, and finally to elimination. The predicted annual accumulation of 0.4 and 0.8 μg/kg of fullerenes and CNTs, respectively, was reported in the sediments accepting the ENM contaminated surface waters (Sun et al., 2014). Due to lack of specific regulations for ENM disposal, CNMs composites are often incinerated, and the formation of aerosols or air borne particles containing the CNTs is possible (Petersen et al., 2011; Nowack et al., 2013). Using mass models, Mueller et al. (2013) proposed that 94% of the CNTs are completely mineralized during the incineration process of waste handling, and that the residual amount discharged to the air and water (waste water treatment plant) is insignificant (<0.0001 and 0.0005%, respectively); 5% is direct disposal into the landfills. Thus, the primary exposure to CNMs could be considered via usage and disposal, rather than during manufacturing processes.

According to life cycle release modeling studies, soils/sediments and landfills are the sink for an estimated 80% of CNMs released into the environment (Keller et al., 2013). Due to their hydrophobicity, CNMs are often readily soluble in organic solvents. However, soil pore water and various other soil components or co-contaminants may affect CNM fate and stability (Bennett et al., 2013). Surfactants and natural organic matter (NOM) have been reported to stabilize CNTs in the aqueous phase, thereby enhancing material mobility in soil systems (Lecoanet et al., 2004; Hyung et al., 2007; Hyung and Kim, 2008; Bennett et al., 2013). Soil properties such as pH, clay and organic carbon content, texture, and mineralogy could affect CNM mobility in the environment (Avanasi et al., 2014). The transport of the CNMs within environmental compartments is dependent on particle colloidal stability as well as their *in situ* transformation. The sorption properties and chemical transformation of CNMs may dictate mobility in soils and sediments, and hence their bioavailability. Zhang et al. (2012) evaluated the association between an aqueous dispersion of MWCNT and different soil minerals (kaolinite, smectite, or shale) under varying sodium levels. Using ^14^C-labeling studies, the authors showed that the removal of MWCNTs from the aqueous phase was directly proportional to the ionic strength and hydrophobicity of the minerals. This shows that the type of soil is a critical factor controlling CNM residence in the pore water that is readily available to both, plants and the soil microbial community. In a related study with C_60_-fullerenes, Avanasi et al. (2014) suggested that CNMs are resistant to mineralization and would persist in soil at least for 1–2 years.

At this point, general conclusions on CNMs fate in the soil environment are difficult to draw as the process is dependent on multiple factors related soil physicochemical characteristics and composition, as well as CNM properties and the identity/susceptibility of potential receptors. Varying physicochemical properties of CNMs produced for specific applications make risk assessment of these particles rather uncertain. Thus, further research on CNM agglomeration, sorption in varying solids and interaction with root exudates and biological fluids is needed before drawing conclusions over CNM fate in the environment. Accurate assessment of fate and associated risks of the CNMs will not only help in regulating the current use of these materials, but also aid in the manufacture of safer-by-design nanomaterials.

## Effects of CNMS in Plants

A number of different carbon-based NMs (CNMs: fullerenes, carbon NPs, fullerol, and SWCNT/MWCNT, among others) have recently gained interest due to their possible applications in regulating plant growth (Khot et al., 2012). Importantly, the literature shows both positive and negative effects on terrestrial plant species, depending upon CNM type and concentration, growth conditions, and plant species. In this section, we present these effects separately.

### Positive Effects

Although most studies have focused on toxicological/physiological endpoints, several early studies did attempt to evaluate CNM accumulation in plants (**Table 1**). In a hydroponic study, Lin and Xing (2007) found significant increase in ryegrass (*Lolium perenne*) root length (∼17%) upon exposure to 2000 mg/L MWCNT (ryegrass) as compared to untreated controls. Canas et al. (2008) evaluated the toxicity of uncoated and coated SWCNTs [poly-3-aminobenzenesulfonic acid (PABS); PABS: *CNT*s = 65:35 (w/w)] to six crop species; cucumber (*Cucumis sativus*), carrot (*Daucus carota*), onion (*Allium cepa*), tomato (*Lycopersicon esculentum*), cabbage (*Brassica oleracea*), and lettuce (*Lactuca sativa*). The plants were exposed hydroponically to coated (0, 160, 900, and 5,000 mg/L) and uncoated-CNTs (0, 104, 315, and 1750 mg/L) for 24 and 48 h. Upon exposure, uncoated-CNTs increased root length in onion and cucumber as compared to the coated-CNTs. Although, variability was high (0–30% for uncoated-CNTs and 5–83% for coated-CNTs), an inverse relationship between exposure time and the extent of root elongation was observed, i.e., 1-day-exposure showed more pronounced effects than 2-day-treatments. Interestingly, microscopic studies revealed no internalization of CNTs into the roots; only surface adsorption was evident. Therefore, authors hypothesized that CNTs might impose indirect effects on plant root systems, such as impeding microbial-root interactions, causing toxicity to microbes or altering crucial biochemical processes such as nutrient acquisition. Wild and Jones (2009) noted that upon exposure to wheat, CNTs were adsorbed onto the root surface but also did appear ‘pierce’ the root epidermal cells and accumulate within the tissue.

**Table 1:** Positive effects of carbon nano-materials (CNMs) in plant.

Reference	CNM	Treatment	Effect
Samaj et al., 2004	CNT	–	Uptake through endocytosis.
Lin and Xing, 2007	MWCNT	2000 mg/L in ryegrass (*Lolium perenne*)	Increased root length (∼17%).
Canas et al., 2008	Uncoated and PABS coated SWCNTs	coated [0, 160, 900, and 5,000 mg/L) and uncoated-CNTs (0, 104, 315, and 1750 mg/L) for 24 and 48 h.	Uncoated-CNTs increased root length in onion and cucumber as compared to the coated-CNTs.
Liu et al., 2009	SWCNT	–	SWNTs as potential cargo for several molecules into different plant cell organelles.
Wild and Jones, 2009	MWCNT	–	CNTs were adsorbed onto the root surface but also did appear ‘pierce’ the root epidermal cells and accumulate within the tissue.
Tripathi et al., 2011	Citrate coated water-soluble CNTs	10-days exposure to 6.0 mg/mL	Visualize internalization of the coated ws-CNTs by SEM and TEM.
Khodakovskaya et al., 2011	SWCNT and MWCNT	50 mg/L	enhanced the total fresh biomass
Mondal et al., 2011	Pristine (diameter ∼30 nm) and oxidized-MWCNT	In mustard (*Brassica juncea*) at 2.3–46.0 μg/L	Enhanced germination, increased root and shoot growth.
Wang et al., 2012	o-MWCNT	40, 80, and 160 mg/L for 3 and 7 days	Increase in root length of wheat seedlings
Sonkar et al., 2012	Water-soluble carbon nano-onions	5 and 10-days hydroponic germination at 10, 20, and 30 mg/L	Growth enhancement.
Lahiani et al., 2013	MWCNT	10–11 d at 50, 100, and 200 mg/L	50% (in barley and soybean) and 90% (in corn) increase in germination. In soybean, the root length increased up to 26%. In corn, shoot and leaf length were enlarged by 40% and more than threefold, respectively. Internalization was visualized by both Raman Spectroscopy and TEM.
Tiwari et al., 2013	MWCNTs	5–60 mg/L MWCNTs for 7 days in agar gel	60 mg/L treatment; increased plant fresh biomass (43%) and higher nutrient uptake (2x calcium and 1.6x iron)
Kole et al., 2013	Fullerols C_60_(OH)_20_	0.943, 4.72, 9.43, 10.88, and 47.2 nM fullerol	Increased plant biomass and phytomedicine content in bitter melon.
Tripathi and Sarkar, 2014	Cabon nano-dots	10 days of exposure to 150 mg/L water soluble carbon nano-dots	Enhanced root growth (10x) of wheat.
Saxena et al., 2014	Water-soluble CNPs	10–150 mg/L ws-CNPs in soil up to 20 days	Optimum growth was observed at 50 mg/L treatment where root and shoot lengths were increased up to 3-times.
Lahiani et al., 2015	Carbon nano-horns (CNHs)	25, 50 and 100 mg/ml for 10–20 days barley, corn, rice, soybean, switchgrass, tomato) and tobacco cell culture	Growth of tobacco cells was increased 78%. Uptake confirmed by TEM.

**Co-contaminants**			

Ma and Wang, 2010	Fullerene + Trichloroethylene (TCE)	2–15 mg/L fullerene in by eastern cottonwood	TCE uptake increase with increase in fullerene concentration.
De La Torre-Roche et al., 2012	Fullerene + DDE	40 mg C_60_ + 100 ng/mL DDE for 3 weeks	Zucchini and soybean, a 29% increase and a 48% decrease in *p,p′*-DDE uptake were observed upon fullerene exposure
Hamdi et al., 2015	CNT + chlordane components; CNT + DDE	1000 mg/L for a 19-day in lettuce	Non-functionalized CNT was more effective at reducing the organochlorine accumulation by plant roots (88%) and shoots (78%).

Conversely, Tripathi et al. (2011) investigated the impact of citrate coated water-soluble CNTs (ws-CNT) in gram (*Cicer arietinum*) after a 10-days exposure to 6.0 mg/mL and were able to visualize internalization of the CNTs by electron microscopy. The authors hypothesized that once present inside the vascular tissue, ws-CNTs formed an ‘aligned network’ that increased water uptake efficiency and directly resulted in the observed plant growth enhancement (Tripathi et al., 2011).

**FIGURE 1:**
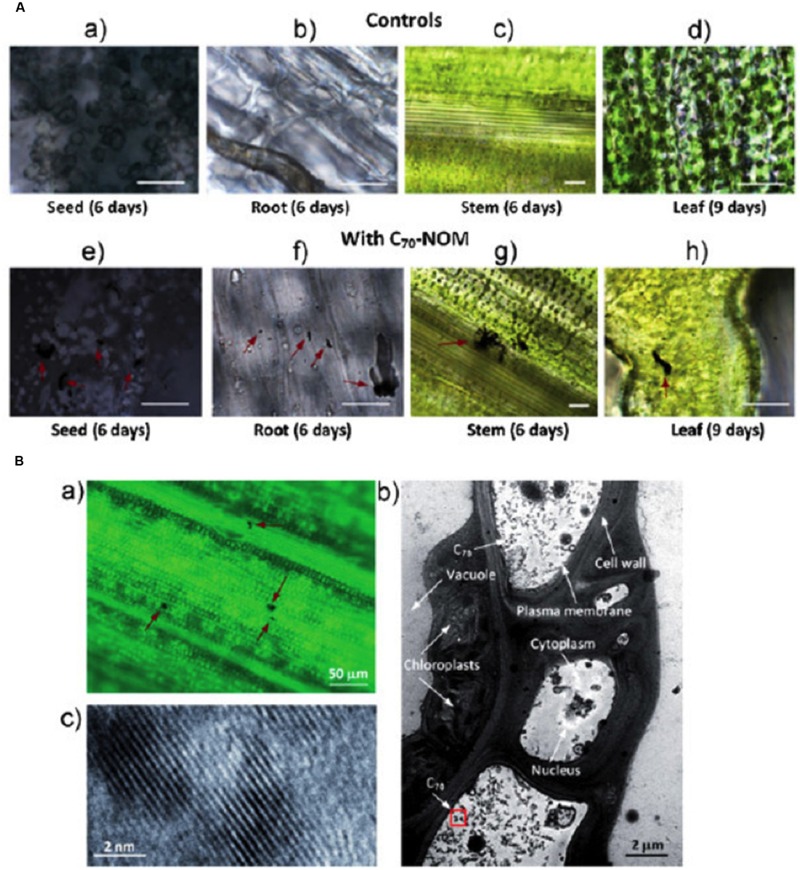
**Bright-field images of rice plants showing C_70_ uptake. (A)** Bright field images of root and leaf portions of 1-week-old rice seedlings. Control plants without any C_70_ (a–d) and treated plants showing C_70_ uptake (e and f). Arrows indicate the aggregation of nanoparticles in corresponding C_70_ treated plant tissues (scale bars are 20 μm). **(B)** (a) Bright field image of the leaf portion of a second generation rice plant. C_70_ aggregates were mostly found near the leaf vascular system. (b) TEM image of the leaf cells showing C_70_ particles (C_70_: 20 mg/L). (c) transmission electron microscopy (TEM) image of C_70_ particles with higher magnification. Reprinted from: Husen and Siddiqi (2014), Copyright © 2014 Husen and Siddiqi; licensee BioMed Central Ltd. [adopted from Lin et al. (2009), Copyright © 2009 Wiley-VCH Verlag GmbH & Co. KGaA, Weinheim].

There have been several other reports of plant growth enhancement upon CNT exposure. Mondal et al. (2011) studied the effect of pristine (diameter ∼30 nm) and oxidized-MWCNT (o-MWCNT; diameter ∼20 nm) on mustard (*B. juncea*) at exposures of 2.3–46.0 μg/L. The authors reported enhanced germination, as well as increased root and shoot growth. At the lowest concentrations, o-MWCNTs yielded higher rates of germination (99% in 22 days) than did the pristine form (94% in 26 days). However, the rate of germination began to decrease at higher MWCNT exposure levels. After 5–10 days of exposure at the lowest concentrations, both root and shoot lengths were increased by 2.5x and 1.6x, respectively, as compared to untreated controls. Similarly, Khodakovskaya et al. (2011) showed that in Murashige and Skoog (MS) growth medium, 50 μg/mL SWCNT and MWCNT exposure enhanced the total fresh biomass of tomato seeds by 75 and 110%, respectively, as compared to activated carbon and graphene. In a follow up study, the authors (Khodakovskaya et al., 2012) compared the effects of MWCNT and activated carbon exposure on tobacco cells and demonstrated that growth was 55–64% higher at 5–500 μg/mL MWCNT exposure as compared to untreated controls. Importantly, although activated carbon enhanced cell growth (16%) at low concentrations (5 μg/mL), growth was suppressed by ∼25% at the higher exposures (100–500 μg/mL). Further investigations from this group revealed an upregulation of several genes upon CNT exposure; including an aquaporin (*NtPIP1*) and two genes, e.g., *CycB* and *NtLRX*, involved in water transport, cell wall formation, and cell division (Khodakovskaya et al., 2012). Similarly, Wang et al. (2012) reported ∼50 and 32% increase in root length of wheat seedlings after 3 and 7 days of exposure to 40–160 μg/L o-MWCNT, respectively. Lahiani et al. (2013) reported the effects of MWCNT exposure (10–11 days at 50, 100, and 200 μg/mL) on the germination and growth of soybean, corn (*Zea mays*), and barley (*Hordeum vulgare*) in agar medium. Upon exposure, nearly 50% (in barley and soybean) and 90% (in corn) increases in germination rate were observed compared to untreated controls. In soybean, the root length increased up to 26% and for corn; shoot and leaf length were enlarged by 40% and more than threefold, respectively. In addition, MWCNTs internalization was visualized by both Raman Spectroscopy and transmission electron microscopy (TEM; **Figure 1**). Similar results were reported by Tiwari et al. (2013) for corn exposed to 5–60 mg/L MWCNTs for 7 days in agar medium. At the 60 mg/L exposure, both plant fresh biomass (43%) and nutrient uptake (2x calcium and 1.6x iron) were increased as compared to controls. Although many studies report positive impacts from CNT exposure, we do note that much of the work is focused on short-term studies with high levels of CNT exposure mostly in artificial growth media. The relevance of these findings to actual agricultural conditions remains unknown but the consistency of the findings across plant species upon CNT treatment clearly warrant further investigation.

Germination and growth enhancement is not limited to CNT exposure; additional CNMs have been shown to positively affect plant physiological parameters as well. Tripathi and Sarkar (2014) reported enhanced root growth (10x) of wheat upon 10 days of exposure to 150 mg/L water soluble carbon nano-dots (ws-CNDs) as compared to controls. Similarly, Sonkar et al. (2012) reported growth enhancement in gram plant from treatment with water-soluble carbon nano-onions (wsCNOs) that were derived from the pyrolysis of wood waste. Specifically, during 5 and 10-days hydroponic germination studies, exposure-dependent growth enhancement was observed at 10, 20, and 30 μg/mL, when compared to untreated controls. In addition to nano-dots and wsCNOs, water-soluble carbon nanoparticle (wsCNP) such as fullerols, a fullerene derivative [C_60_(OH)_20_], is another candidate for carbon-based plant growth enhancement. Kole et al., 2013 observed that fullerol increased both the biomass and phytomedicinal content of bitter melon (*Momordica charantia*), which is a source of various compounds used in the treatment of diseases such as AIDS, diabetes, and cancer (Ng et al., 1992; Raman and Lau, 1996; Basch et al., 2003). Specifically, plants treated with 0.943, 4.72, 9.43, 10.88, and 47.2 nM fullerol exhibited a 54 and 128% increase in biomass and fruit yield, respectively. In the fruit tissue, fullerol exposure significantly increased the content of cucurbitacin-B (74%) and lycopene (82%), both of which are anticancer compounds, and the antidiabetic molecules Charantin and insulin were increased by 20 and 91%, respectively (Kole et al., 2013). Saxena et al. (2014) examined the concentration dependent effects of water-soluble CNPs (ws-CNPs) on wheat. The ws-CNPs were isolated from naturally occurring raw CNPs present in biochar. Plants were treated with 10-150 mg/L ws-CNPs in soil for up to 20 days; results showed optimum growth at 50 mg/L treatment with root and shoot lengths increased up to three times compared to untreated controls.

Carbon nano-horns (CNHs) have also been reported positively impact the growth of terrestrial plants (Lahiani et al., 2015). CNHs are spherical structures with “disordered single-layered graphene sheets with a lateral size of up to 10 nm and an interlayer distance of approximately 4–5 Å” (Xu et al., 2011). Lahiani et al. (2015) exposed tobacco cells to CNHs at 25, 50, and 100 μg/ml for 24 h and noted a 78% increase in growth of cultured tobacco cells at 100 μg/ml while no significant effects at 25 μg/ml, as compared to controls. Recently, Zhang et al. (2015) treated tomato seeds in cotton-cushioned glass bottles with 40 μg/ml graphene. Upon exposure, the germination rate at 2, 4, and 6 days was increased by 26.6, 43.4, and 13.5%, respectively, when compared to untreated controls.

**FIGURE 2:**
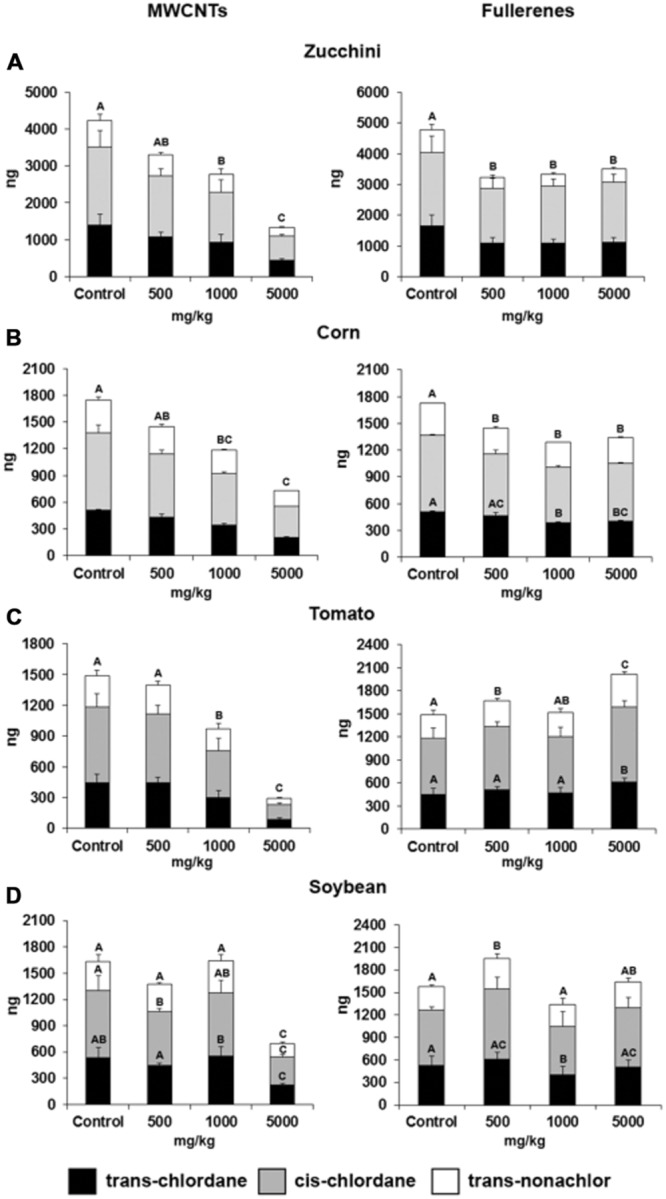
**Total plant content of chlordane components in soil-grown **(A)** zucchini, **(B)** corn, **(C)** tomato, and **(D)** soybean co-exposed to 0–5000 mg/kg MWCNT or C_60_ fullerene.** Error bars are the standard error. Within a plant species and nanomaterial type, chlordane components with different letters are significant different. If no letter is shown, differences are not significant. Reprinted with permission from De La Torre-Roche et al. (2013), Copyright © 2013 American Chemical Society.

Apart from direct positive impacts on physiology and growth, select CNMs have been shown to have significant impacts on the fate and transport of several organic co-contaminants. For example, Ma and Wang (2010) reported fullerene-dependent uptake of trichloroethylene (TCE) by eastern cottonwood (*Populus deltoides*); increases were 26 and 82% at 2 and 15 mg/L fullerene, respectively. Conversely, De La Torre-Roche et al. (2012) investigated the effects of C_60_ fullerene on the bioaccumulation of *p,p′*-DDE by zucchini, soybean, and tomato in vermiculite and observed differential uptake of *p,p′*-DDE across different plant species. In zucchini and soybean, a 29% increase and a 48% decrease in *p,p′*-DDE uptake were reported upon fullerene exposure, respectively, but no effect was evidenced in tomato. In a follow up soil-based study, the accumulation of weathered chlordane and DDx (DDT + metabolites) decreased by 21–80% across four crops (zucchini, corn, tomato, and soybean) upon MWCNT co-exposure to 500–5000 mg/kg (De La Torre-Roche et al., 2013; **Figure 2**). However, C_60_ co-exposure exhibited mixed effects, ranging from increased chlordane uptake by 34.9% (soybean/tomato) to complete loss of DDx uptake (tomato/corn). Species dependent uptake of DDE was also observed by Hamdi et al. (2015), where the effects of CNTs surface modification on the uptake of chlordane components (*cis*- and *trans*-chlordane, *trans*-non-achlor) and *p,p′*-DDE by lettuce was evaluated. After exposure to 1000 mg/L for 19 days, non-functionalized CNT reduced organochlorine content in plant roots and shoots by 88 and 78%, respectively, but reductions were significantly higher with amino-functionalized CNT (root: 57% and shoot: 23%).

From the above discussion, it is clear that CNMs have potential to enhance plant growth, nutrient uptake, seed germination, and fruit quality. Among the CNMs, CNTs are the most extensively studied and have shown promising positive effects, with low to moderately high doses of CNTs improving overall plant growth. CNTs and fullerenes were also found to have the secondary positive effect of reducing the accumulation of pesticides by select plant species. However, across all studies the beneficial responses are largely dependent on plant species, nature of the growth medium, CNM type/concentration, and growth conditions.

### Negative Effects

A review of the literature reveals a number of reports of showing adverse effects on plants from exposure to a range of CNMs (**Table 2**). Similar to the positive effects, toxicity was found to be largely dependent on CNM concentrations, growth/exposure conditions, and plant species. However, a general lack of soil based studies confound efforts to extrapolate these findings to field conditions. Results from some of the representative studies are discussed below.

**Table 2:** Negative effects of CNMs in plant.

Reference	CNM	Treatment	Effect
Stampoulis et al., 2009	MWCNT	Zucchini for 15-day exposure to 1000 mg/L	60% reduction in biomass reduction.
Mondal et al., 2011	Oxidized-MWCNT	Hydroponic mustard	Reduced germination and dry biomass.
Liu et al., 2013	ws-C_70_	Tobacco BY-2 cells were exposed to 0.01 mg/mL ws-C_70_ for 3 days in cell culture medium.	Cell boundary disruption and growth inhibition. Possible adsorption of ws-C70 to the cell wall through hydrostatic interaction with the carboxylic groups of fullerenes.
Begum et al., 2011	Water-soluble graphene oxide (ws-GO)	Lettuce, cabbage, Red spinach, and tomato during a 20-days exposure	At 2000 mg/L, significantly reduced plant growth (up to 78%), biomass (up to 88%), the number and size of leaves (up to 53 and 91%, respectively), and increased ROS along with necrotic symptoms.
Anjum et al., 2013	Graphene oxide (GO)	*Vicia faba* beans 100–1600 mg/L	Concentration dependent decrease in oxidative enzyme activity.
Anjum et al., 2014	Graphene oxide	*Vicia faba* beans 100–1600 mg/L	Highest concentration (1600 mg/L) resulted in growth reduction, decreased anti-oxidative enzyme activity (e.g., catalase and ascorbate peroxidase), and greater electrolyte leakage.

**Co-contaminants**			

Hu et al., 2014	GO + Arsenic	0.1–10mg/L GO	Arsenic-GO co-exposure significantly reduced the fresh mass, shoot length, and chlorophyll content.

Stampoulis et al. (2009) investigated the effect of MWCNT exposure under hydroponic conditions on zucchini. Upon 15-day exposure to 1000 mg/L, a 60% reduction in biomass reduction was observed when compared to control and bulk carbon. A separate hydroponic study by Mondal et al. (2011) revealed dose dependent toxicity of MWCNTs in mustard, where oxidized-MWCNT exerted more negative effects than pristine MWCNTs. At ‘high’ exposure concentration, both pristine (46 mg/L) and oxidized-MWCNT (6.9 mg/L) caused toxicity, reducing germination by 4.4 and 7.6% and dry biomass by 1.6 and 2.2-fold, respectively, as compared to the lowest concentration. Begum et al. (2011) investigated the species-dependent toxicity of wter-soluble graphene (GO; ‘graphene oxide (GO) with sodium ions as the counter-ions’) in lettuce, cabbage, red spinach, and tomato during a 20-days exposure period. At the highest concentration (2000 mg/L), graphene significantly reduced plant growth (up to 78%), biomass (up to 88%), reduced the number and size of leaves (up to 53 and 91%, respectively), and increased reactive oxygen species (ROS) production and necrotic symptoms in all plants except lettuce. In an *in vitro* study, *Arabidopsis thaliana* (Columbia ecotype) T87 cells grown in Jouanneau and Péaud-Lenoel (JPL) media were exposed to 0–80 mg/L graphene (Begum and Fugetsu, 2013). Significant increase in fragmented nuclei, membrane damage, ROS generation, mitochondrial dysfunction, and induced cell death were observed upon exposure. Moreover, Anjum et al. (2013) evaluated the effect of GO on the fava bean (*Vicia faba*) glutathione redox system, a major determinant of cellular redox homeostasis. Concentration dependent stress-response (order: 1600 > 200 > 100 mg/L GO), as well as decreased oxidative enzyme activity were observed. In a follow up study, Anjum et al. (2014) reported no toxicity below 800 mg/L of GO; the highest concentration (1600 mg/L) resulted in growth reduction, decreased anti-oxidative enzyme activity (e.g., catalase and ascorbate peroxidase), and greater electrolyte leakage.

Apart from CNTs and GOs, there are reports of fullerene toxicity to higher terrestrial plants. Liu et al. (2013) studied the effects of water-soluble carboxyfullerenes [ws-C_70_; C_70_(C(COOH)_2_)_2-4_] in tobacco BY-2 cells (*Nicotiana tobacum*, cv. Bright Yellow). Here, BY-2 cells were exposed to 0.01 mg/mL ws-C_70_ for 3 days in cell culture medium. The results showed cell boundary disruption and growth inhibition, possibly due to the adsorption of ws-C_70_ to the cell wall through hydrostatic interaction with the carboxylic groups of fullerenes. In a co-exposure study with GO and arsenate [As(V)], Hu et al. (2014) found that 0.1–10 mg/L GO exposure enhanced the adverse effects of As(V) in wheat seeds, significantly reducing the fresh mass, shoot length, and chlorophyll content of treated plants. In addition, the activity of peroxidase (POD) and superoxide dismutase (SOD), likely biomarkers for stress response, were increased in a concentration-dependent manner.

In summary, the negative effects exerted by the CNMs are specific to growth conditions and plant species. However, the limited and contradictory reports in the literature confounds the efforts to make consistent generalized observations with regard to CNM exposure. Clearly more research needs to be done to uncover the mechanisms of CNM-plant interactions and these fundamental studies should be conducted under a range of growth conditions and with a large number of plant species. Only then can one generalize/quantify potential negative effects in a way that would ensure safe wide-scale application of CNM under field conditions.

## Effects of CNMS on Plant-Associated Soil Microbes

**FIGURE 3:**
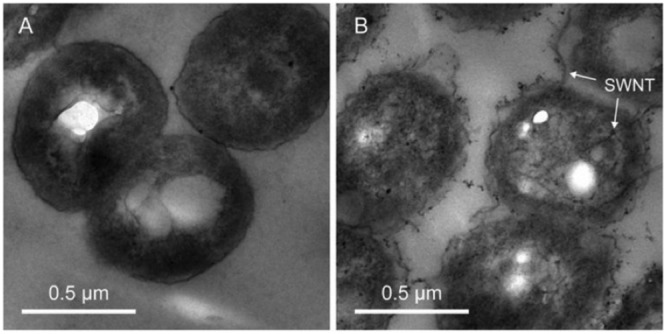
**Transmission electron microscopyTEM images of *Paracoccus denitrificans* cells in the absence **(A)** and presence of 50 mg/L carboxyl-modified SWNT **(B)** after 24 h of exposure.** Zheng et al. (2014), Nature Publishing Group, a division of Macmillan Publishers Limited.

**FIGURE 4:**
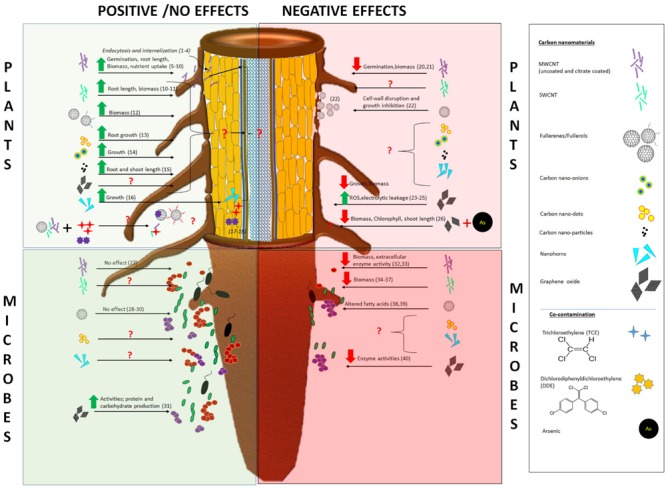
**Uptake, internalization, and effects of carbon nanomaterials on plants and plant associated soil microbes.** References: (1) Samaj et al. (2004); (2) Liu et al. (2009); (3) Wild and Jones (2009); (4) Tripathi et al. (2011); (5) Lin and Xing (2007); (6) Mondal et al. (2011); (7) Wang et al. (2012); (8) Lahiani et al. (2015); (9) Tiwari et al. (2013); (10) Khodakovskaya et al. (2009, 2011); (11) Canas et al. (2008); (12) Kole et al. (2013); (13) Tripathi and Sarkar (2014); (14) Sonkar et al. (2012); (15) Saxena et al. (2014); (16) Lahiani et al. (2015); (17) Ma and Wang (2010); (18) De La Torre-Roche et al. (2012); (19) Hamdi et al. (2015); (20) Stampoulis et al. (2009); (21) Mondal et al. (2011); (22) Liu et al. (2013); (23) Begum et al. (2011); (24) Anjum et al. (2013); (25) Anjum et al. (2014); (26) Hu et al. (2014); (27) Shrestha et al. (2013); (28) Tong et al. (2007); (29) Nyberg et al. (2008); (30) Cordeiro et al. (2014); (31) Wang et al. (2013); (32) Chung et al. (2011); (33) Kerfahi et al. (2015); (34) Liu et al. (2009); (35) Kang et al. (2007); (36) Rodrigues et al. (2013); (37) Jin et al. (2014); (38) Fang et al. (2007); (39) Johansen et al. (2008); (40) Chung et al. (2015).

Soil microbial communities have a direct impact on soil quality through processes such as nutrient cycling, decomposition of organic matter, and symbiotic relationships with terrestrial plant species (Kennedy and Smith, 1995). Therefore, protection of soil microbial biomass and diversity is a major challenge in agriculture. Currently, limited information is available on the interaction between CNTs and soil microbial community (Simonet and Valcarcel, 2009; Dinesh et al., 2012). CNMs may be directly toxic to soil microorganisms (**Figure 3**), may alter the bioavailability of nutrients, or may increase or reduce the toxicity of organic compounds and/or toxins (Dinesh et al., 2012). In addition, toxicity to plants may indirectly impact microbial communities. In this section, we review the current literature on the interactions of CNT with soil microbes; similar to the section on plants, we divide coverage of work into positive and negative impacts. **Figure 4** summarizes the positive and negative impacts of the CNMs on the plants and the plant associated soil- microorganisms.

### Positive Effects

Although limited information is available, unlike plants, CNMs seem to be generally toxic to soil microbes (**Table 3**). Nevertheless, a few studies have revealed neutral or positive biological effects in soil microorganisms. For example, the impact of fullerenes (C_60_) on soil microbial community populations was evaluated using total phospholipid derived phosphate (Tong et al., 2007). Soil was treated with 1 and 1000 μg C_60_/g soil for 180 days; the results showed that fullerenes had no impact on the structure or function of the soil microbial community or on soil enzymatic activities (Tong et al., 2007). Similarly, Nyberg et al. (2008) exposed anaerobic wastewater treatment sludge to fullerene (C_60_) concentrations up to 50,000 mg/kg and showed no significant effects on microbial community activity after several months. Shrestha et al. (2013) reported diverse effects on soil (sandy loam) microbial communities after 90 days of exposure to MWCNT. The authors utilized a wide range of CNT concentrations (10–10,000 mg/kg); at lower concentrations (10, 100, and 1000 mg/kg), no observable effects were evidenced on soil-microbial composition and enzymatic activities. However, at 10,000 mg/kg a mixed response was observed; decreased abundance was detected in select bacterial species (e.g., *Waddlia, Holophaga, Derxia, and Opitutus*). Notably, the amount of polycyclic aromatic hydrocarbon (PAH) degrading organisms (e.g., *Cellulomonas, Rhodococcus, Pseudomonas*, and *Nocardioides*) was markedly increased. These results suggest a potential shift toward more stress tolerant organisms with increasing soil-MWCNT concentration, although the findings are too limited to be conclusive. Additionally, Wang et al. (2013) showed that GO exposure at 0.1 g/L enhanced the activity of anaerobic ammonium-oxidizing bacteria by 10%. The authors reported a dose-dependent enhanced production of protein and carbohydrate with GO concentrations of 0.05–0.1 mg/mL (Wang et al., 2013). Although not related to plant species, Cordeiro et al. (2014) studied six bacterial colonies (Gram-negative) isolated from the mucus of the estuarine worm *Laeonereis acuta* (Nereididae), and showed no changes in the growth of the colonies after 24 h exposure to aqueous fullerene (aq-C_60_) suspensions at 0.01, 0.10, and 1.00 mg/L. The mechanisms responsible for these interactions are unknown, however, this limited literature does suggest that under certain exposure scenarios, CNM hay have neutral or perhaps modestly beneficial effects on microbial communities.

**Table 3:** Positive/Neutral effects of CNMs in soil microorganism.

Reference	CNM	Organism	Treatment	Effect
Tong et al., 2007	Fullerene (C_60_)	Microbial communities	1 μg and 1000 μg C_60_ /g	No impact on the structure or function and enzymatic activities.
Nyberg et al., 2008	Fullerene (C_60_)	Microbial communities	Up to 50,000 mg/kg	No significant effects on microbial community activity.
Shrestha et al., 2013	MWCNT	Microbial communities	10–100 mg/kg	No observable effects on soil-microbial composition and enzymatic activities at lower concentrations.
			10,000 mg/kg	Decreased abundance in select bacterial species.
Wang et al., 2013	Graphene oxide	Anaerobic ammonium-oxidizing bacteria	0.1 g/L	Enhanced activity of anaerobic ammonium-oxidizing bacteria by 10%.
			0.05–0.1 mg/ml	Enhanced production of protein and carbohydrate.
Cordeiro et al., 2014	Fullerene (C_60_)	Gram-negative bacterial colonies	0.01, 0.10, and 1.00 mg/L	No changes in the growth of the colonies after 24 h exposure.

### Negative Effects

Although the mechanism of CNT toxicity is not well understood, possible antimicrobial activity has been observed (**Table 4**). It is evident that CNTs interact strongly with bacteria cell membranes; these strong electrostatic interactions may disrupt membrane structure integrity by oxidative stress and/or physical damage (puncture of membrane; Jackson et al., 2013). For example, studies with a range of bacteria (gram-negative *Escherichia coli, Pseudomonas aeruginosa*, and gram-positive *Staphylococcus aureus, Bacillus subtilis*) evaluated the toxicity of SWCNT dispersed (5 g/mL) in Tween 20 saline solution in comparison with SWCNT aggregates in saline solution. Results showed a higher antibacterial activity of dispersed SWCNT to gram-positive bacteria. The SWCNTs dispersed in Tween 20 saline solution showed antibacterial activity of 58.1 ± 5.0% for *E. coli*, 65.1 ± 1.8% for *P. aeruginosa*, 87.5 ± 6.5% for *B. subtilis*, and 85.6 ± 5.3% for *S. aureus*. On the other hand, SWCNT-aggregates in saline solution showed reduced antibacterial activity of 33.8 ± 4.0% for *E. coli*, 27.7 ± 5.9% for *P. aeruginosa*, 53.9 ± 2.8% for *B. subtilis*, and 50.3 ± 3.5% for *S. aureus*. The authors reported that the enhanced antimicrobial activity was due to the smaller overall size and higher mobility in solution when compared with aggregates, which resulted in greater physical damage to bacterial membranes (Liu et al., 2009). Also, the antimicrobial activity of SWCNT has been reported on bacteria deposited on surfaces and in suspension (Jackson et al., 2013). Tong et al. (2012) quantitatively investigated the effects of surface coating of SWCNTs on soil microbial community under low and high organic matter concentration. Upon 6000 μg/g of functionalized SWNTs (fSWCNT; coated with polyethylene glycol or m-polyaminobenzene sulfonic acid) exposure for 6 weeks, researchers observed some microbial community shift keeping the total biomass unaffected. Studies with purified SWNTs (less than 0.8 wt% cobalt) at concentrations varying from 1 to 50 μg/mL exhibited strong antimicrobial properties to *E. coli.* Results indicated that direct contact of SWNT aggregates with *E. coli* cells caused loss of viability and subsequent inactivation. The authors also reported that toxicity was dependent on incubation/contact time with the SWNTs; the average of losses in viability were 73.1 ± 5.4%, 79.9 ± 9.8%, and 87.6 ± 4.7% at 30, 60, and 120 min, respectively (Kang et al., 2007). Another important factor that may influence the toxicity of CNTs to bacteria is the presence of residual impurities. Commercial CNTs are synthesized with strong acids and contain up to 4.5–15% of metals such as cobalt (Co), iron (Fe), nickel (Ni), and yttrium (Y) and other impurities, which may exert toxic effects on microbes (Kang et al., 2007; Petersen et al., 2014).

**Table 4:** Negative effects of CNMs in soil microorganism.

Reference	CNM	Organism	Treatment	Effect
Kang et al., 2007	SWNTs	*Escherichia coli*	1–50 μg/mL	Strong antimicrobial properties.
Fang et al., 2007	C_60_ aggregates	*Bacillus subtilis* (Gram-positive) and *Pseudomonas putida* (Gram-negative)	0.01 mg/L	Significantly impacted levels of iso-and anteiso-branched fatty acids in *Bacillus subtilis* (Gram-positive).
Johansen et al., 2008	Fullerenes C_60_	Microbial communities and protozoans	0–50 mg/kg	Results after 14 days showed a threefold decrease in the number of fast-growing bacteria.
				No significant changes in protozoan population.
Liu et al., 2009	SWCNT dispersed and SWCNT agglomerates in saline solution	Gram-negative *Escherichia coli, Pseudomonas aeruginosa*, and gram-positive *Staphylococcus aureus, Bacillus subtilis*	5 g/mL	Higher antibacterial activity of dispersed SCNT to gram-positive bacteria in comparison with the agglomerates.
Chung et al., 2011	MWCNT	Microbial communities	0, 50, 500, and 5000 μg/g	Enzyme activity decreased after 30 min of the incubation.
				Microbial biomass and extracellular enzyme activity decreased (up to ∼50%).
Rodrigues et al., 2013	Carboxyl-functionalized SWCNTs	Bacterial and fungal communities	0.5 mg/L	Alteration on *Pseudomonas putida* (Gram-negative) phase transition temperatures and levels of unsaturated fatty acids.
				Higher doses had a maximum biomass loss at 3 days and the fungal community was unable to recover even after 14 days.
Jin et al., 2014	SWCNT	Gram- positive, Gram-negative bacteria, and fungal populations	0.03 to 1 mg/g	Decreased biomass of microbial groups and fungal populations.
Chung et al., 2015	Graphene oxide	Microbial communities/soil enzymes	0.5–1 mg/kg	Decreases of up to 50% in the enzyme activities after 21 days of incubation.
Kerfahi et al., 2015	Raw and acid treated or functionalized MWCNTs	Microbial communities	0–5000 mg/kg	Bacterial community composition was affected but recovered after 8 weeks.

Another possible mechanism of CNT toxicity is the induction of ROS, which may then directly interact with organelles to induce DNA damage or protein inactivation that results in apoptosis and cell death (Jackson et al., 2013). Additionally, others have reported diverse toxic effects of carbon-based materials on soil bacteria. For example, Johansen et al. (2008) evaluated protozoans and bacterial total respiration, biomass, and diversity upon exposure to C_60_ fullerenes at 0–50 mg/kg dry soil. Results after 14 days showed a threefold decrease in the number of fast-growing bacteria (appearing 3 days after plating) after C_60_ exposure; while the protozoan population was only slightly (but not significantly) affected at the highest fullerene concentration (Johansen et al., 2008).

Fang et al. (2007) showed that C_60_ aggregates in water at 0.01 mg/L significantly increased the levels of iso-and anteiso-branched fatty acids from 5.8 to 31.5% and 12.9 to 32.3% in *B. subtilis* (Gram-positive), suggesting an increase in membrane fluidity as an adaptation response to C_60_. Alternatively, the aq-C_60_ at 0.5 mg/L resulted in an alteration on *P. putida*’s (Gram-negative) phase transition temperatures and levels of unsaturated fatty acids from bacterial membrane. Rodrigues et al. (2013) reported the effect of carboxyl-functionalized SWCNTs on soil bacterial and fungal communities. Soil was amended with 0, 250, and 500 mg/kg functionalized SWCNT for 14 days and the populations were monitored over time. The authors reported that after 3 days, the number of colony-forming units (CFUs) was significantly decreased but that the population had recovered after 14 days. Alternatively, higher doses of SWNTs had a similar biomass loss at 3 days but the fungal community was unable to recover even after 14 days. Similarly, Jin et al. (2014) reported that at relatively low concentrations (0.03–1 mg/g), the biomass of gram-positive and gram-negative bacteria, as well as fungal populations, showed a negative correlation with SWCNT concentration in soil. Additionally, in a 3-week study, the authors reported that SWCNTs showed similar toxicological responses to MWCNTs but at five-times lower concentrations due to the higher surface area of single wall tubes (Jin et al., 2013). Chung et al. (2011) investigated the effect of MWCNT on the microbial communities treated with 0, 50, 500, and 5000 μg/g MWCNT in two different soil types (sandy loam and loamy sand) for 30 min, 1, 4, and 11 days. The enzyme activity decreased at 30 min of incubation and for most enzymes, the suppression persisted until day 11. In both soils, microbial biomass and extracellular enzyme activity decreased (up to ∼50%) and reductions were more notable at higher exposure concentrations (500 and 5000 μg/g). Recently, Kerfahi et al. (2015) investigated the effects of native and functionalized MWCNTs (0–5000 mg/kg) on soil bacterial. The authors reported that at 2 weeks, the soil bacterial community composition was affected by the fMWCNT at the highest concentrations; however, after 8 weeks there was no effect on the bacterial diversity with either type of nanotube. The authors attributed this early effect to the acidic nature of fMWCNTs, which caused a decrease in soil pH at higher exposure concentrations and subsequently changed (temporarily) soil bacterial communities (Kerfahi et al., 2015). Chung et al. (2015) studied the impact of GO at 0.5–1 mg/kg and noted a decrease up to 50% in the activity of select key soil enzymes, including xylosidase, 1,4-ß-N-acetyl glucosaminidase and phosphatase after 21 days of exposure. Clearly, the published literature suggests that CNMs may have a significant negative effect on soil microbial communities (**Figure 4**). However, there is limited information on the broader impacts of these adverse effects, including implications for symbiotic or co-habitating terrestrial plant species. **Figure 4** also highlights the gaps in the literature with respect to uptake and internalization of the CNMs, as well as their effects on the soil biota.

## Limitations and Future Perspective

### Non-Environmentally Relevant Growth Conditions

Considering the varied applications of CNTs and their scope of use in agriculture, it is necessary to design realistic exposure scenarios to investigate CNM fate and effects. This includes testing environmentally realistic concentrations under relevant environmental conditions. In looking at the existing literature, it is clear that for obvious reasons, neither of these parameters have been met. To date, there is still lack of knowledge on the amount of nanomaterials released into the environment. Modeling studies have predicted that CNT concentrations in waste water treatment plant eﬄuent in the San Francisco Bay area are approximately 0.01–0.05 μg/L, while in dry biosolids the levels range from 0.05 to 0.1 μg/kg (Keller et al., 2013). Gottschalk et al. (2009) used a model-based on probabilistic material flow analysis to estimate the CNT concentrations in US sludge-treated soils were 0.4 μg/kg. Other studies have predicted the environmental concentration of additional NMs (Gottschalk et al., 2009); these estimates coincide with the range of predicted concentrations for CNTs in the environment. Importantly, the vast majority of the existing nanotoxicology literature has used exposure concentrations that are few orders of magnitude above than predicted to currently exist. For example, commonly used concentrations in soil studies range from 10 to 5,000 mg/kg (De La Torre-Roche et al., 2013; Shrestha et al., 2013), while in hydroponic studies concentrations vary from 2.3 μg/L to 5000 mg/L (Canas et al., 2008; Mondal et al., 2011). Although, such ‘high’ exposure concentrations are useful for comparative analysis of inherent NM toxicity, the data is often inappropriate for broader estimations of actual risk. Another major confounding factor in the current literature is the general lack of soil-based studies with regard to CNT fate and effects. The complex interactions of CNT with NOMs and/or pollutants in soil are noted above and can have significant effects on material fate, behavior, and toxicity in the environment. From analytical perspective, it is also very difficult to quantify CNMs at very low levels (μg/kg or μg/L) under high background concentration of organic carbon and other organic/inorganic compounds coming from NOMs. Moreover, sample collection, transportation, preservation can produce significant artifacts on CNMs. Additionally, many published studies use “neat” or “pristine” CNTs, which fails to recognize the importance of weathering and transformation in soil, as well as the fact that in most applications, CNTs will be a component of a larger and more complex polymer or formulation. In fact, only a few studies have used acid-treated CNT that are commonly used in industry and are expected to be present in the environment where interactions with soil and biota will occur (Kerfahi et al., 2015). Since laboratory based recent studies will likely lead to inaccurate predictions of CNT fate and effects, assessment under “realistic” conditions and in more complex and relevant biological scenarios is greatly needed to appropriately assess the risk from the widespread CNT in agricultural settings.

### Lack of Mechanistic Understanding

Not surprisingly, our review of the existing literature has revealed that majority of research on CNM-toxicity is focused on straight-forward evaluation of physiological and biochemical effects. The interaction among various environmental parameters (growth media, exposure time, and receptor/species differences) and CNMs (type, synthesis conditions, and concentrations) are complex and efforts to ascertain consistent or broad trends with regard to material fate and effects is difficult. In addition, artifacts arising from sample preparation, storage, and/or experimental design can confound results and lead to biased or inaccurate data interpretation (Petersen et al., 2014). Importantly, little progress has been made on understanding the underlying mechanisms of toxicity at the molecular/genetic level. The reasons for the lack of study at this scale are numerous and include the: (i) current technological limitations to detecting CNMs in complex growth media, (ii) unavailability of *in situ* real-time monitoring techniques to track CNMs inside the living tissues and different environmental components, (iii) complex, heterogeneous, and inconsistent nature of the soil matrix, (iv) lack of knowledge of the distribution of CNMs in the environment, and (v) species and growth condition dependency of the CNM induced toxicity. Additionally, in the growth media, during exposure, the NM surface properties may get altered, which can subsequently cause changes in material bioavailability and toxicological outcomes (Mukherjee et al., 2014a,b; Servin et al., 2015). On the other side, the impact of agricultural feedstock on nanomaterial synthesis, structure, and reactivity still remains elusive. There are few reports where organic extracts from agricultural plants, e.g., grass and tea, among others, has been successfully used as the alternative carbon sources for CNM green-synthesis *in-situ* (Ruan et al., 2011; Jacob et al., 2015). Nonetheless, the mechanistic understanding of this bottom-up approach remains mostly unknown and needs further investigation.

Three major modes of action for NMs interaction are known: (i) dissolution (Bandyopadhyay et al., 2015), (ii) direct contact (Zhao et al., 2013, 2014), and (iii) co-transport of other contaminants (De La Torre-Roche et al., 2012, 2013; Lynch et al., 2014). Pristine CNMs are resistant to dissolution (Garner et al., 2015); however, functionalized CNMs such as carboxylated-CNTs and C_60_-OH are much more likely to dissolve in the growth media (Kole et al., 2013; Saxena et al., 2014). Toxic heavy metals (e.g., Ni, Co, and Fe) associated with CNMs (mostly CNTs) could also alter the overall toxicological profile (Liu et al., 2007). Therefore, a comprehensive knowledge of the dissolution/degradation kinetics of the CNMs in the environment is necessary to understand the underlying mechanistic pathways. Additionally, direct physical contact has been shown to alter plant physiological and biochemical parameters such as biomass production, growth, germination rates, and ROS production. Last, there are several studies in the literature demonstrating CNM mediated alteration of pesticide uptake/toxicity in terrestrial plants, including zucchini, soybean, tomato, lettuce etc. (De La Torre-Roche et al., 2012; Hamdi et al., 2015).

Unlike metallic NMs, there are very few robust analytical methods available for detecting CNM in the environment (Petersen et al., 2011; Bandyopadhyay et al., 2012a,b, 2013; Guo et al., 2015). The environmentally relevant concentration of CNMs can go down to as low as 0.05 μg/kg (Keller et al., 2013). Researchers use various analytical techniques, e.g., TEM, SEM, AFM, and Raman spectroscopy, among others, to determine the presence of CNMs inside the plant tissue. These techniques are capable of identifying compounds up to sub-nanometer level. However, imaging techniques, e.g., TEM and SEM need extensive sample preparation (e.g., solid-phase extraction, liquid-liquid extraction, fixation, staining, etc.), which can alter the morphology of individual CNMs or their clusters (Petersen et al., 2014). Moreover, softer imaging techniques, e.g., Raman spectroscopy (with high wavelength laser) might lead to surface modification of the CNMs. Therefore, technological limitation is clearly evident and much focus needs to be directed toward the development of imaging technologies with very little or no prior sample preparation without compromising the sub-nanometer resolution.

## Conclusion

From the above discussion, it is clear that a general lack of understanding persists for CNM fate and effects in the environment. The somewhat limited literature that does exist is mixed for most species, with both positive and negative effects being observed. The reasons for these mixed effects are numerous (different exposure scenarios, growth conditions, particle type/concentration, and species, among others). As such, reliable and accurate assessment of risk, which is necessary before widespread application of CNMs in agriculture, is not possible with the current knowledge base. Only comprehensive investigations of chronic exposure under environmentally realistic scenarios will enable such efforts. Future research should also focus on molecular/genetic-level studies under environmentally relevant conditions. Information gained from whole genome/proteome/metabolome analyses of different organisms (model organism or crop species) could prove to be a powerful resource for assessing the risks of CNM-exposure and potentially shedding light on the key factors involved in the mechanisms of cellular response to uptake and storage. Effects of CNMs on soil bacterial populations (in terms of modification in relative abundance of different species or isolation of hypersensitive/tolerant strains), coupled with more efficient characterization of the interactions between CNMs and organic/inorganic matter present in soil, can add further information in a more relevant context of a biological “realistic” scenario. This will inherently increase the complexity of the studies and, at the same time, decrease the gap between experimental and environmental conditions.

## Author Contributions

AM, SM, AS, LP, OD, and JW collected data from the literature and drafted the manuscript. All the authors wrote and revised the article.

## Conflict of Interest Statement

The authors declare that the research was conducted in the absence of any commercial or financial relationships that could be construed as a potential conflict of interest.
